# Seaweed Secondary Metabolites In Vitro and In Vivo Anticancer Activity

**DOI:** 10.3390/md16110410

**Published:** 2018-10-26

**Authors:** Djenisa H. A. Rocha, Ana M. L. Seca, Diana C. G. A. Pinto

**Affiliations:** 1 Department of Chemistry & QOPNA-Organic Chemistry, Natural Products and Food Stuffs, University of Aveiro, Campus de Santiago, 3810-193 Aveiro, Portugal; djenisa@ua.pt (D.H.A.R.); ana.ml.seca@uac.pt (A.M.L.S.); 2 cE3c-Centre for Ecology, Evolution and Environmental Changes, Azorean Biodiversity Group & Faculty of Sciences and Technology, University of Azores, Rua Mãe de Deus, 9501-321 Ponta Delgada, Portugal

**Keywords:** seaweeds, secondary metabolites, cytotoxic activity, cancer, terpenoids, bromophenols, dictyolactone, cholest-5-en-3β,7α-diol, halomon, laurebiphenyl

## Abstract

Isolation, finding or discovery of novel anticancer agents is very important for cancer treatment, and seaweeds are one of the largest producers of chemically active metabolites with valuable cytotoxic properties, and therefore can be used as new chemotherapeutic agents or source of inspiration to develop new ones. Identification of the more potent and selective anticancer components isolated from brown, green and red seaweeds, as well as studies of their mode of action is very attractive and constitute a small but relevant progress for pharmacological applications. Several researchers have carried out in vitro and in vivo studies in various cell lines and have disclosed the active metabolites among the terpenoids, including carotenoids, polyphenols and alkaloids that can be found in seaweeds. In this review the type of metabolites and their cytotoxic or antiproliferative effects will be discussed additionally their mode of action, structure-activity relationship and selectivity will also be revealed. The diterpene dictyolactone, the sterol cholest-5-en-3β,7α-diol and the halogenated monoterpene halomon are among the reported compounds, the ones that present sub-micromolar cytotoxicity. Additionally, one dimeric sesquiterpene of the cyclolaurane-type, three bromophenols and one halogenated monoterpene should be emphasized because they exhibit half maximal inhibitory concentration (IC_50_) values between 1–5 µM against several cell lines.

## 1. Introduction

Cancer is one of the deadliest diseases, and its influence on European and USA mortality is 20% and 14% respectively [1]. It is estimated that in 2018 the numbers will reach values of 18 million new cases and 10 million deaths [1] and it is expected that, in the near future, this numbers will increase [2]. Lung cancer is the most common, both in terms of incidence and mortality, followed by female breast and colorectal cancers. These types of cancers account, each, for nearly 2 million diagnoses in 2018 [1,3]. The top three more frequent cancers diagnosed in women are the breast, lung and colorectal cancers [3], being the lung cancer responsible for more deaths. Whereas in men lung cancer is also the leading cause of death and the more frequent cancer diagnosed, followed by prostate and colorectal cancers [1].

Although significant advances are being made, fully effective cancer therapy is a far way from being achieved [4,5,6]. The cancer heterogeneity [7], and the development of resistance to anticancer drugs [8] constitute one of the major problems to be overcome. In fact, the treatment of cancer usually comprises a combination of therapies, in accordance with the characteristics and stage of the tumor, including surgery, radio and/or chemotherapy and most recently immunotherapy [9,10,11]. Thus, currently, the development of drugs for a specific cancer-related target, combined with an effective understanding of the drug relationship with human tumor biology became the key in the effort to cure cancer [12].

One of the main sources of drugs are natural compounds, which have demonstrated considerable potential in the cancer therapy [13,14]. In fact, at least one-third of the current top twenty drugs are derived from natural sources, including plants and marine species, and among the 175 small molecules approved to treat cancer, 49% are either natural compounds or directly derived therefrom [15].

Seaweeds or macroalgae are photosynthetic organisms that play a key role in ocean biodiversity and productivity and comprise green algae (Chlorophyta), brown algae (Phaeophyta) and red algae (Rhodophyta). Furthermore, seaweeds are also a source of unique secondary metabolites that showed very interesting bioactivities [16,17,18,19,20]. Due to its nutritional value, seaweeds have also been used as food in many countries of East Asia (Japan, Korea, and China) and in the Celtic cultures of Europe (Ireland, Scotland and Brittany), and used as additive in cosmetic and food industries [21,22].

In the last two decades, seaweed chemical profiles have demonstrated that they are rich in terpenoids, alkaloids, polyphenols, steroids, pigments and polysaccharides and some biological assays showed that several of these metabolites have promising pharmacological activities [23,24,25,26] including in cancer therapy [27,28,29].

This review aims to highlight the secondary metabolites isolated from seaweeds with the highest cytotoxic/antiproliferative activity. Their activity level, chemical structures, putative mechanisms of action will be discussed. Moreover, the reported in vivo studies will be emphasized. It is important to highlight that this is not an exhaustive review but rather an author’s selection, where the compounds whose biological activity and mechanism of action suggest that they have significant therapeutic potential. Thus, the discussed and cited studies involve seaweed secondary metabolites exhibiting cytotoxic activities lower than 15 µM. Exceptions can also be mentioned if they are rare cases or the mechanism of action is elucidated.

## 2. Secondary Metabolites from Seaweeds with in Vitro Cytotoxic Activity

### 2.1. Mono-, Sesqui-, Diterpenes and Sterols

Epidemiological and experimental studies suggest that terpenoids may be helpful to curb the growth of a variety of cancer cells, including mammary, skin, lung, forestomach, colon, pancreatic and prostate carcinomas cells and open more opportunities for cancer therapy [30,31].

**Figure 1:**
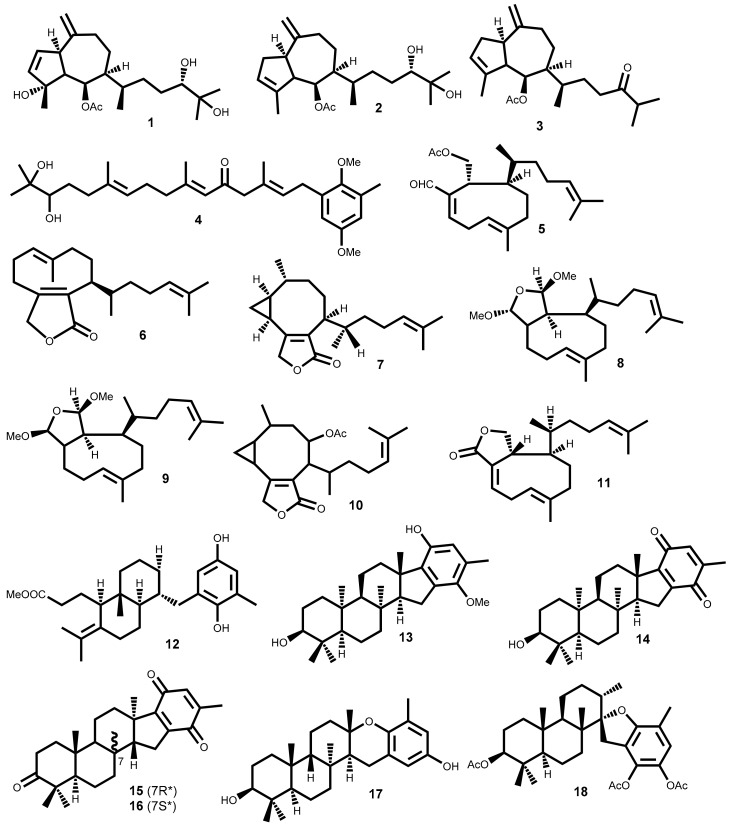

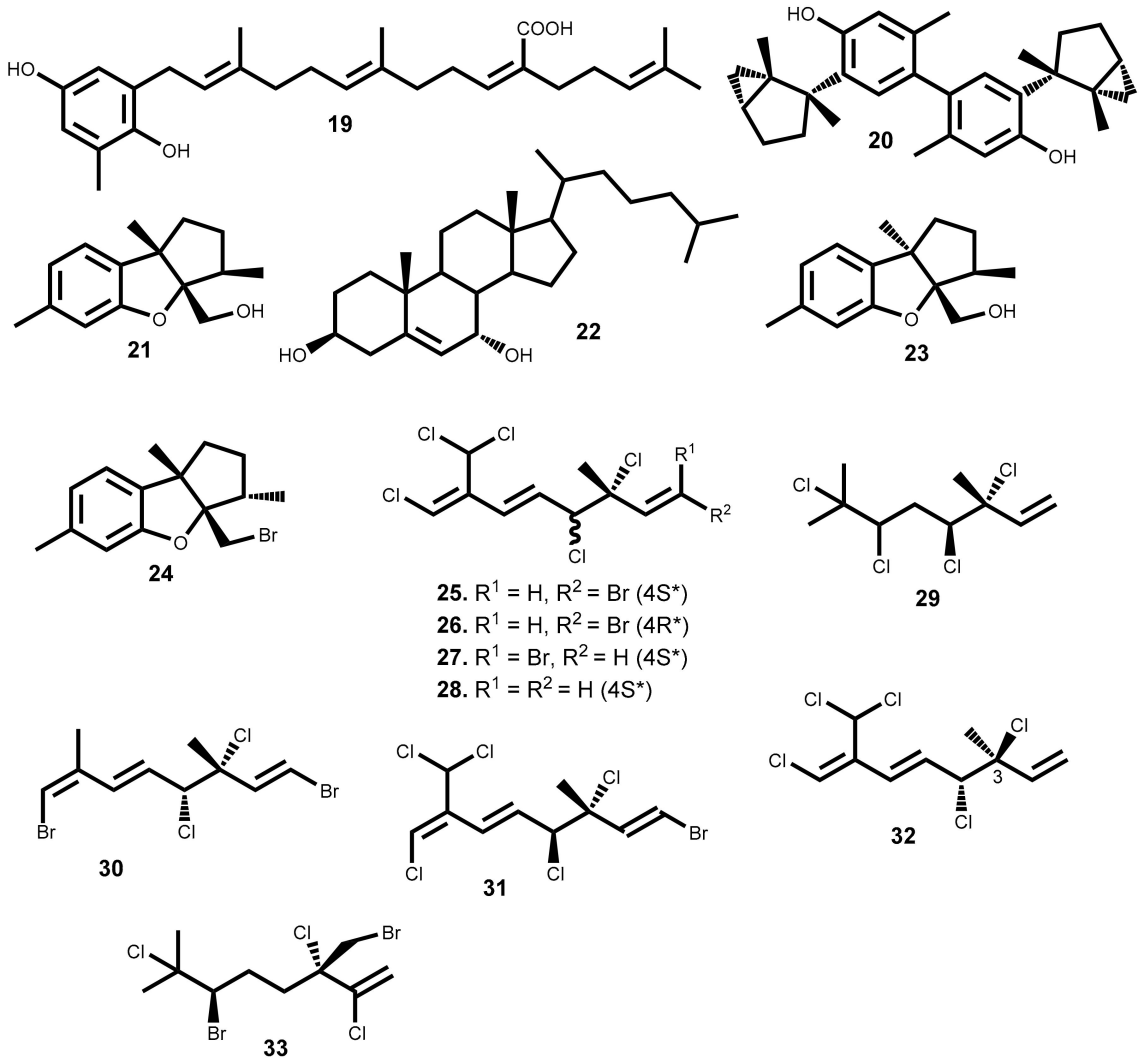
Chemical structure of the terpenoids **1**–**33** isolated from seaweeds that presented significant cytotoxic activity.

The search for anticancer terpenoids from seaweeds in the last 30 years resulted in a significant number of compounds with relevant cytotoxic activity against a large number of cancer cell lines, highlighted on Table 1 and whose chemical structures are presented in Figure 1.

From the 33 compounds shown in Table 1, 19 were isolated from brown seaweeds, whereas the remaining were isolated from red seaweeds. One third exhibit moderate activity (10 μM < IC_50_ <15 μM) against the cancer cell lines tested, while nearly 20 compounds exhibit significant cytotoxic activity, with IC_50_ values between 1 and 10 μM, at least against one of the cancer cell line tested. Among the latter, compounds **20** (laurebiphenyl) and **31** [(1*E*,5*E*,7*E*)-3,4-*erythro*-1-bromo--7-dichromethyl-3-methyl-3,4,8-trichlorooctatriene] can be emphasized, as they exhibit IC_50_ values between 1–5 µM (Table 1).

The detailed analysis of the results compiled in Table 1 allowed choosing compound **22** (cholest-5-en-3β,7α-diol) as the most interesting one, due to the lower IC**_50_** values (<0.5 μM) against several cell lines. Additionally, also exhibited significant activity (IC_50_ values 5–9 µM) against gastric and colon cancer lines. Compounds **11** (dictyolactone) and **33** (halomon) also should be highlighted due to their strong activity (IC_50_ = 0.99 and 0.92 µM, respectively) against human non-small cell lung carcinoma cells (NSCLCN6-L16) and colon cancer cell line (HCT-116).

Unfortunately, very few compounds (only 6 out of 33) were subjected to selectivity studies and the more active ones, mentioned above, are not included in this lot. Debromoplysinol (**23**) and isoaplysin (**24**) are the most selective compounds, exhibiting cancer cell selectivity index of 4.1 and 3.8, respectively, which means nearly four times more selective to the prostate carcinoma cells than to mammary epithelial no-tumoral cells.

The results obtained in cytotoxic evaluations of hydroazulene skeleton type diterpenes isolated from the marine brown alga *Polycladia myrica* (S.G. Gmelin) Draima, Ballesteros, F. Rousseau & T. Thibaut (syn. *Cystoseira myrica* (S.G. Gmelin) C. Agardh), where compounds **1**–**3** (Figure 1) were included, suggest that the presence of free hydroxyl groups is irrelevant [32].

Cytotoxicity of several meroditerpenes in different phases of the HeLa cell line growth cycle conducted by Gouveia et al. demonstrated that the HeLa cell line in log phase is 3.6 times more sensitive to cystoazorol A **4** than in lag phase [34]. Moreover, compound **4** showed a selectivity index higher than taxol, compound used as a positive control in the study [34].

From the Mediterranean brown algae *Dilophus ligulatus* (Kützing) Feldmann, which is the second genus that provides more compounds with interesting cytotoxic activity, can be emphasized the diterpenoids **5**–**11** that exhibit IC_50_ values lower than 15 µM (Table 1). For example, murine leukemia cells (P-388) and P-388 doxorubicin resistant cell lines exhibit similar sensitivity (IC_50_ = 3.64 to 13.3 µM and IC_50_ = 5.95 to 12.9 µM, respectively) to diterpenes **6**–**9** and **11** [35]. Furthermore, compounds **6**–**11** exhibit higher cytotoxic activity against NSCLCN6-L16 (IC_50_ 0.99–6.85 μM) than mercaptopurine (IC_50_ 6.57–19.7 μM), an effective anticancer drug used as positive control [35].

Both flabellinol **13** and flabellinone **14** (Figure 1), two polycyclic diterpenoids fused to an oxidized ring and biosynthetically related, were cytotoxic against NCI-H460 (IC_50_ 9 and 14 μM, respectively). The action mechanism is related with their ability to block the sodium channel activity [37]. From the brown algae *Taonia atomaria* (Woodward) J. Agardh collected in Central Aegean Sea were isolated two cyclic meroditerpenoids, atomarianone A **15** and atomarianone B **16**, that are epimers at C-7 and both are analogues of flabellinone **14** (Figure 1). The different relative configuration at C-10 suggests that they are formed in a different diterpene cyclization pathway, nevertheless both exhibit significant cytotoxicity against two different lung cancer cell lines (Table 1) [38].

The antiproliferative properties of six meroditerpenoids were evaluated against human and non-human cancer cell lines and against no-tumoral cell line by Pereira et al. [39]. Epitaondiol **17** and stypotriol triacetate **18** (Figure 1) were the most active compounds, being the thrice-cloned neuroblastoma SH-SY5Y the most susceptible cell line tested (IC_50_ 12.2 and 14 µM, respectively), though much less active than vincristine (IC_50_ = 0.03 µM) used as positive control [39]. Furthermore, compounds **17** and **18** have lower selective index than other compounds tested, once at 25 µM have more than 50% of proliferation inhibition on non-cancer Chinese hamster fibroblasts cell line (V79) [39].

The red alga of the genus *Laurencia* is collected in different parts of the world, but typically inhabit tropical oceans and are recognized for the biosynthesis of a high diversity of secondary metabolites, especially terpenoids mainly sesquiterpenoids, sterols and acetogennins. Some of these metabolites exhibit important pharmacological effects, from which cytotoxic can be highlighted [54,55,56]. From the compounds indicated in Table 1, five (**20**–**24**) were isolated from *Laurencia* species, the third genus with more compounds presented in Table 1 (after the genera *Plocamium* and *Dilophus*). Among the compounds isolated, the most active one is cholest-5-en-3β,7α-diol **22**, the ones with the broadest action spectrum are laurebiphenyl **20**, cholest-5-en-3β,7α-diol **22** and debromoaplysinol **23**, and the most selective one is debromoaplysinol **23** (Table 1).

Isoaplysin **24** and debromoaplysinol **23** are two sesquiterpenes that differ in the presence of the C3a-hydroxymethyl instead of the C3a-bromomethyl moiety (Figure 1). Compound **23** displays enhanced selectivity and broad spectrum cytotoxicity relative to **24** which, according to the authors, suggests that the hydroxyl group could play a significant role in the cytotoxicity of this class of compounds [45]. However, these compounds also have different configuration around the C3 and C8b chiral centers and that could explain the activity displayed. In fact, it is also observed that the stereochemistry of the C8b chiral center can play an important role in the drug-ligand interactions. This is well demonstrated by the strong activity of debromoaplysinol **23** when compared with debromoepiaplysinol **21** (Figure 1, Table 1). Compound **21** was tested against 5 cancer cell lines (lung, hepatic, cervix, gastric and ileum cancer cell lines) and shown to be much less active (IC_50_ values greater than 15 µM) than compound **23** [44].

*Plocamium* is the genus with greater representativeness in Table 1 (8 secondary metabolites). The six halogenated monoterpenes **25**–**30** (Figure 1) isolated from the red macroalga *Plocamium suhrii* Kützing collected near Port Elizabeth, South Africa, showed great cytotoxicity (IC_50_ < 10 µM) against esophageal cancer cell line (WHCO1), even higher than the well-known clinical anticancer drug cisplatin (IC_50_ = 13 µM) [47]. Moreover, the low variation of the cytotoxicity level of compounds **25**–**30** against WHCO1 cell line (IC_50_ = 6.6 to 9.9 µM) shows that the C1 double bond configuration (comparing compound **25** and **27**), the C4 chiral center configuration (comparing compounds **25** and **26**), and the presence of bromine atoms in the structure (comparing compounds **27** and **28**), have little or no effect on the compounds’ cytotoxicity.

Last year, was reported the isolation of halogenated monoterpenes **25**, **31** and **32** from the red alga *Plocamium cartilagineum* (Linnaeus) P.S.Dixon (syn. *Plocamium cartilagineum*) collected in South Africa [48]. Additionally, the compounds’ cytotoxic effects were evaluated and stereoisomers **25** and **31** exhibit identical and significant cytotoxicity against colon cancer cell line (IC_50_ = 3.3 µM) and low effect against lung cancer cell line (NCI-H460) [48]. In the attempt to find the possible mechanism of action was confirmed that none of the compounds have activity as sodium channel blockers or activators [48].

Halomon [6(*R*)-bromo-3(*S*)-bromomethyl)-7-methyl-2,3,7-trichloro-1-octene] **33** (Figure 1), an halogenated monoterpene isolated for the first time from *Portieria hornemannii* (Lyngbye) P. C. Silva, showed high cytotoxic activity against renal-, brain-, colon-derived solid tumor cell lines while leukaemia and melanoma cells are less sensitive to this compound [53]. This interesting case of differential cytotoxicity led this compound for in vivo and preclinical drug development. Unfortunately, several constraints such as low and variable natural content, and poor in vivo results, has hampered the clinical development of this compound. Conversely, the high interest of scientific community in the isolation of enough halomon **33** material lead to the reinvestigation of *Portieria hornemannii* (Lyngbye) P. C. Silva and to the consequent isolate of other halogenated monoterpenes. This fact allowed some structure/activity relationship studies that demonstrated no crucial role of the halogen at C7 neither the C6 nor C7 hybridization to the activity while the halogen atoms at C6 and C2 are crucial for the high cytotoxicity [57]. The mechanism of action of halomon-type monoterpenes have not been completely elucidated but it was demonstrated that halomon **33** possess potent inhibitory activity of the DNA methyltransferase-1 (DNMT-1) (IC_50_ = 1.25 µM), an enzyme responsible for methylation of cytosine residues residing at CpG sites. In fact, in many cancers, promoters of tumor suppressor genes are silenced by hypermethylation at CpG sites, and thus, the inhibition of DNMT-1 could potentially reverse tumor growth [58]. In truth, the interest in halomon **33** never fainted; the first total synthesis was achieved in 1998, halomon **33** was obtained in 13 steps with an overall yield of 13% [59] and in 2000 Sotokawa et al. described the synthesis of the racemic halomon, in three-steps (25%) and poor selectivity [60]. Fifteen years later, the first selective method was described, which was also efficient [61]. Recently, halomon was synthesized using the previous method with some improvements in the enantioselectivity resulting also in preparation of several halomon-analogues and halogenated natural products [62]. Thus, conditions are created to advance with the clinical studies and/or development of new anticancer drugs based on the halomon scaffold.

**Figure 2:**
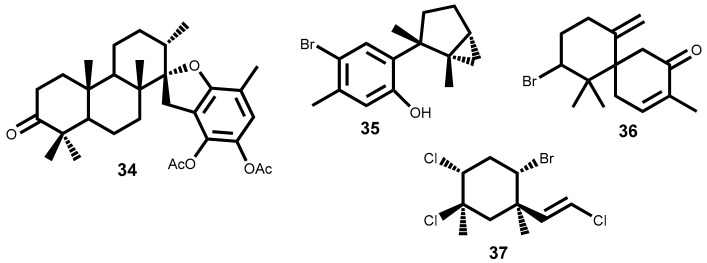
Seaweed terpenoids, **34**–**37**, with relevant studies on its cytotoxic action mechanism.

Apart from the few cases mentioned above, the study of the compounds action mechanisms is vague. However, some studies on the mechanism of action of terpenoids extracted from seaweeds which are not indicated in Table 1 can be found in the literature. This means that the compounds showed lower activity or the IC_50_ value has not been reported. Nevertheless, information about possible mechanisms of action is important to understand the action of natural sesquiterpene. One example is the 14-keto-stypodiol diacetate **34** (Figure 2), a diterpenoid isolated from the seaweed *Stypopodium flabelliforme* with great structural similarity with compound **18** (Figure 1), whose cytotoxic effect on prostatic cancer cell proliferation (DU-145, IC_50_ = 24 µM), and its action mechanism was studied [63]. 14-Keto-stypodiol diacetate **34** acts on microtubule assembly inhibition, induces mitotic arrest and has strong inhibition effect on plasminogen activator secretion [63]. These events are all correlated and affect the cell invasive capacity [64,65]. Moreover, the identification of the signaling pathway influenced by the microtubule cytoskeleton [66] may offer a source of novel anticancer treatments.

Laurinterol **35** (Figure 2), is a marine sesquiterpene isolated from a red alga *Laurencia (Laurencia okamurae* Yamada) [55], *L. pacifica* [45]*, Laurencia nidifica* J. Agardh [67] that structurally is very similar to a part of laurebiphenyl **20** (Figure 1). Laurinterol **35** was reported for the first time as potential anticancer metabolite against melanoma cells (B16F1) with IC_50_ value of 33.9 µM. and several studies were applied to understand its mechanism of action in B16F1 cells [55]. The results showed that **35** can induce apoptosis via activation of p53 transcription factor in melanoma cells and also increases the cell numbers in sub-G1 phase by DNA fragmentation [55].

Dactylone **36** (Figure 2), a natural marine halogenated sesquiterpenoid isolated from *Laurencia glandulifera* (Kützing) Kützing [68] was most recently evaluated for its effect on the mouse skin epidermal JB6P^+^Cl41 [69]. The dactylone **36** inhibits epidermal growth factor-induced transformation in JB6P^+^Cl41 cells, induces G1-S cell progression arrest and apoptosis of tumor cells. Also, decreases the expression of cyclin D3 and Cdk4 and cause the suppression of phosphorylation of the Rb protein of JB6P^+^Cl41 cells at Ser^780^, Ser^807/811^ and Ser^795^ in a dose-dependent manner. Similar inhibitory effects on phenotype expression were observed in the human tumor SK-MEL-28, HCT-116 and H460 cell lines [69]. Although this study didn’t present the IC_50_ values, they should, in our opinion, be presented herein because the cancer-preventive properties and some details of the dactylone **36** molecular mechanism of action can be important to understand the action of other natural sesquiterpene.

The last example, is mertensene **37** (Figure 2), a polyhalogenated monoterpene isolated from the red alga *Pterocladiella capillacea* (S.G. Gmelin) Santelices & Hommersand, that inhibit the viability of two human colorectal adenocarcinoma cell lines HT29 [70]. The results showed that mertensene **37** effect is correlated with the activation of mitogen-activated protein kinase (MAPK) extracellular signal–regulated kinase (ERK)-1/-2, serine/threonine-specific protein kinase (Akt) and nuclear factor-kappa B (NF-_K_B) pathways. In cell cycle mertensene-induced G2/M arrest which was associated with a decrease in the phosphorylated form of the anti-tumor transcription factor *p*53, retinoblastoma protein (Rb), cdc2 and chkp2. In apoptosis, mertensene triggers a caspase-dependent apoptosis by activation of caspase-3, the cleavage of poly (ADP-ribose) polymerase (PARP) and increased expression level of death receptor-associated protein TRADD [70].

The results discussed above confirm the role of terpenoids isolated from different species of brown and red alga as potential cancer chemotherapeutic and/or chemopreventive agents, against a large panel of cancer cells. However, deeper research to understand structure/activity relationships and to evaluate their cytotoxicity in no-tumoral cells is necessary. Furthermore, the most promising isolated metabolites should be evaluated in in vivo studies.

### 2.2. Carotenoid

**Figure 3:**
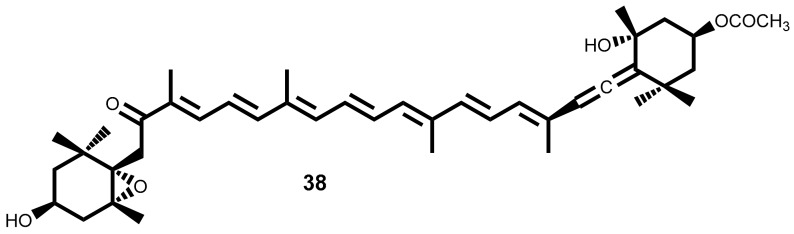
Chemical structure of fucoxanthin isolated from seaweeds.

Fucoxanthin **38** (Figure 3) is an orange-colored pigment tetraterpenoid belonging to xanthophylls subclass of carotenoids, that contributes to more than 10% of the total of carotenoids in nature, and being brown seaweeds their richest source in marine ecosystems [71,72,73]. Some of the examples of macroalgae that are excellent sources of this carotenoid are *Undaria pinnatifida* (Harvey) Suringar [74,75], *Ectocarpus siliculosus* (Dillwyn) Lyngbye [73], *Saccharina japonica* (Areschoug) C.E. Lane, C. Mayes, Druehl & G.W. Saunders (syn *Laminaria japonica* Areschoug) [76], *Sargassum fulvellum* (Turner) C. Agardh and *Hizikia fusiformis* (Harvey) Okamura [72].

The beneficial effects of fucoxanthin are widely described in the literature and are scientifically proven. In fact, the fucoxanthin have a very broad application ranging from prevention to treatment of cardiovascular diseases, oxidative stress, diabetes, obesity, hypertension, anti-inflammatory [20,24,25,77,78,79]. It also exhibits cytotoxic and anti-proliferative effects against a large number of cancer cell lines [80,81,82,83]. Fucoxanthin is clearly a great candidate for chemopreventive and/or chemotherapeutic use in the cancer battle. This can be concluded from the excellent review published by Satomi [83], where the antitumor and cancer-preventive effects of fucoxanthin are discussed and systematized. Herein we resume the main experimental evidences of fucoxanthin cytotoxic and/or antiproliferative effects (Table 2) as well as the main accepted wisdom about fucoxanthin mechanisms of action in different cancer cell lines.

The studies summarized in Table 2 suggest that fucoxanthin exerts its antiproliferative and cancer-preventing effects by modulating the expression of various cellular molecules and cellular signal transduction pathways, being the most cited mechanisms: (i) induction of G1 cell-cycle arrest, independent of the apoptosis induction. Involving also, one or more factors, such as the increase of p15^INK4B^, p21^WAF1/CIP1^ and p27^KIP1^ and connexin 32/43 expression, the activation of MAPK pathways, the decrease of myelocytomatosis (MYCN) proto-oncogene expression and suppression of NF-ĸB activity; (ii) induces a caspase dependent apoptosis accompanied by an increase of reactive oxygen species (ROS) generation and mitochondrial membrane permeability while occur decrease in B-cell lymphoma-extra large (BCL-xL), cellular inhibitors of apoptosis protein (CIAP)1, CIAP2, X-linked inhibitor of apoptosis protein (XIAP) and surviving proteins level, reduction in phosphoinositide 3-kinase (PI3K) and AKT activity and downregulation of signal transducer and activator of transcription 3 (STAT3)/epidermal growth factor receptor (EGFR ) signaling; (iii) induces metastasis inhibition by decrease the matrix metallopeptidase 9, cluster of differentiation 44 (CD44) and CXCR4.

In addition to the wide effects of fucoxanthin **38**, the toxicological studies conducted in male and female mice showed that single doses (1000 or 2000 mg/kg) or repeated treatment (500 or 1000 mg/kg for 30 days) of this pigment, was neither toxic nor promotes histological alteration in mice [101].

The research involving fucoxanthin **38** is currently more focused on its use in combination therapy for the treatment of leukemia, aiming to solve adverse side effects such as the development of resistance and effects on no-tumor cells [102]. Other recent studies involve the development of new delivery systems such as encapsulation-based delivery systems [103,104] and combination with nanoparticles [105,106]. The objective of these new approaches is to overcome fucoxanthin **38** poor in vivo properties, such as low bioavailability and low chemical stability.

It is recognized that fucoxanthin **38** is metabolized to fucoxanthinol in the gastrointestinal tract by digestive enzymes, such as lipase and cholesterol esterase and then absorbed into intestinal cells [107]. Thus, fucoxanthinol seems to be the in vivo bioactive compound [82]. In fact, some authors studied the fucoxanthinol activity and described its ability to induces apoptosis on human colon adenocarcinoma cell line (CaCo-2), human breast adenocarcinoma cell line (MCF-7), human leukemia cell line (HL-60), human adenocarcinoma breast cell line (MDA-MB-231) [108,109], to promote antiproliferative effects on PC3 and T cell leukemia [110,111] and to induce apoptosis in body-cavity-based lymphoma cell line (BCBL-1) and primary effusion lymphoma cell line (TY-1) cells and G1 cell cycle arrest [112].

### 2.3. Phenolic Compounds

Phenolic compounds are composed of a single aromatic ring bearing one or more hydroxyl group to a polymeric structure of this simple unit and exhibit a large broad of biological activities [113,114,115]. The most common subclasses of polyphenols in seaweeds are halogenated phenols, catechins, flavonols, and phlorotannins, being this last more common in brown seaweed while bromophenols, polyphenolics compounds with one or more bromine substituents, are most commonly found in red seaweeds [28,113,116,117]. Polyphenols presence and concentrations in seaweeds can be linked to environmental factors [118,119], but is also dependent of the seaweed species [120,121].

#### 2.3.1. Phlorotannins

**Figure 4:**
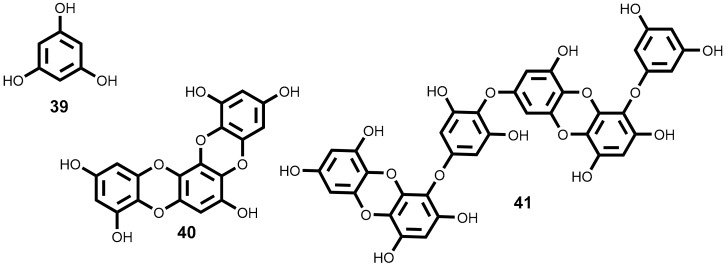
Chemical structure of phloroglucinol **39** and phlorotannins **40** and **41** isolated from seaweeds.

Phlorotannins are described as polyphenolic secondary metabolites formed by polymerization of phloroglucinol **39** (Figure 4) monomer units highly hydrophilic and with a wide range of molecular sizes [113,122]. Based on their interlinkage, phlorotannins can be classified in subclasses, such as phlorethols, fucols, fucophlorethols, eckols, fuhalols and carmalols [122,123,124].

There is evidence that phlorotannins and its derivatives play important roles as anticancer metabolites, acting in different hallmarks of cancer such as proliferative signaling, metastasis, cell cycle, resistance to cell death, evasion, angiogenesis and evasion of growth suppressors. These metabolites can also be considered as chemopreventive agents due to their antioxidant effect, once oxidative stress may act in cancer initiation, promotion and progression [113,117,125,126].

From the brown seaweeds *Ecklonia Cava* Kjellman [127] and *Ecklonia stolonifera* Okamura [128] were isolated several phloroglucinol derivatives. One of them, dioxinodehydroeckol **40** (Figure 4), showed moderate antiproliferative activity against breast cancer cell lines, MCF-7 and MDA-MB-231, being MCF-7 more sensitive than MDA-MB-231. Dioxinodehydroeckol **40** inhibited 53% of MCF-7 cells proliferation at 10 µM [127] and induced the cells’ apoptosis through NF-κB dependent pathway, by an increase in the caspases-3 and -9 activities and cleavage of PARP, accompanied by activation of p53 and Bax and Bcl-2 inhibition [127]. The phlorotannin **40** (Figure 4), also exhibits protective activity against ultraviolet radiation B (UVB)-induced apoptosis of HaCaT cells (treatment of HaCaT cells post-UVB exposure with compound **40** allowed 72% less of cell death). Apparently, phlorotannin **40** can modulate the Bax/Bcl-2 and caspases expression [129]. This is an interesting result because indicates its potential to protect skin against UVB effects.

Dieckol **41** (Figure 4), the major component of *Ecklonia Cava* Kjellman [127], was described as an anti-metastatic compound, having the ability to regulate the metastasis-related genes action on MCF-7 cells, inhibiting the expression of MMP-9 (matrix metalloproteinase-9) and VEGF (vascular endothelial growth factor), and stimulated the expression of TIMP-1 and -2 (tissue inhibitor of metalloproteinase) [130]. This phlorotannin also exhibits a significant pro-apoptotic activity on the ovarian cancer cell line (SKOV3) associated with ROS production. The action mechanisms studied demonstrate that dieckol **41** triggered the activation of caspase-3, -8 and -9 via ROS production and the regulation of AKT and p38 signalling [131].

Phloroglucinol **39** (Figure 4), although is the base-unit of phlorotannins, was also assayed against breast cancer (MCF7, SKBR3 and BT549) stem-like cells which are largely responsible for return of breast cancer [132]. The authors demonstrated that treatment with phloroglucinol **39** suppresses sphere forming ability and anchorage-independent colony formation in breast cancer cells by decreasing the expression of Sox2, CD44, Oct4, Notch2 and β-catenin, the cancer stem-like cell regulators. Moreover, treatment with compound **39** sensitized breast cancer cells to anticancer drugs such as cisplatin, etoposide, and taxol as well as to ionizing radiation. This work proposed that phloroglucinol prevents disease relapse [132]. In the same year, the authors published another study highlighting the antimetastatic effects of phloroglucinol **39** on breast cancer cells (BT549 and MDA-MB-231) through inhibition of the PI3K/AKT and the rat sarcoma (RAS)/RAF-1/ERK mitogenic pathways thereby suppressing epithelial-mesenchymal cell transition [133].

#### 2.3.2. Bromophenols

**Figure 5:**
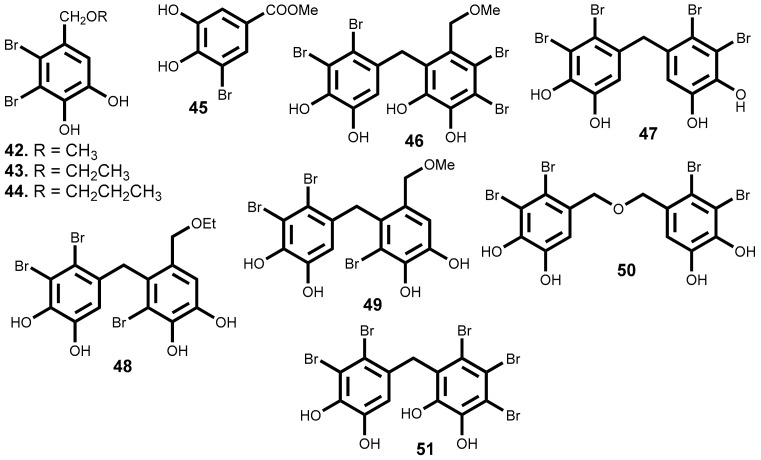
Chemical structure of bromophenols **42**–**51** isolated from seaweeds.

Since, the isolation of the first bromophenols from red algae *Neorhodomela larix* (Turner) Masuda (syn. *Rhodomela larix* (Turner) C. Agardh) [134], many novel bromophenols were isolated [135,136,137,138,139] and some of them, as it is indicated in Table 3, are very interesting cytotoxic compounds. The most interesting features will be discussed below and their structures are depicted in Figure 5.

The literature review carried out and summarized in Table 3 shows that there are 10 compounds belonging to the subclass of the bromophenols that exhibit cytotoxic activity with IC_50_ ≤ 15 µM. Among these, mention should be made to compounds **46**, **48**, and **50** for their relevant cytotoxicity (IC_50_ ≤ 9 µM) against a wide range of at least 6 cancer cell lines, and a particular emphasis to compound **51**, which exhibits the highest cytotoxic activity against the six cancer cell lines, lung, gastric, breast, melanoma, ovarian and colon cell lines (IC_50_ ≤ 3.8 µM) (Figure 5 and Table 3). Compound **47** exhibit identical cytotoxic activity but only against 4 tumor cell lines (IC_50_ ≤ 3.8 µM against lung, gastric, breast and colon cancer cell lines). Compound **44** is the one with the lowest IC_50_ value in Table 3 (IC_50_ = 1.32 µM) against a colon cancer cell line, whereas compounds’ **51** and **47** present the lower values against a lung cancer cell line (IC_50_ = 1.6 and 1.8 µM respectively).

Unfortunately, from the works cited in Table 3 only the work performed by Lijun et al. evaluates the compounds selectivity [141]. They test the compounds, under the same experimental procedure, against the human embryo lung fibroblasts cell line.

The report of the values obtained with positive controls (anticancer drugs in clinical use) and evaluate in the same conditions is also rare. As far as we could find only Shoeib et al. report such data [137], however we should emphasize that the absence of these data significantly reduces the impact of the published results. The compounds **43** and **44** exhibit higher cytotoxic activity against HCT-116 (IC_50_ = 2.51 and 1.32 μM, respectively) than the 5-fluorouracil (IC_50_ = 4.93 μM), an effective anticancer drug used as positive control. On the other hand, HCT-116 cell line is 5-fold and 9-fold, respectively, more sensitive to the compounds **43** (ethyl ether lanosol) and **46** (*n*-propyl ether lanosol) than to compound **42** (methyl ether lanosol) while the human colon adenocarcinoma cell line (DLD-1) is much less sensitive to any of these compounds. Studies of structure-activity relationship showed that the activity is influenced by the number and position of bromine atoms, as well as the number of hydroxyl groups and aliphatic side chain [137].

Wu et al. [145] described the inhibitory effects of compound **47**, bis (2,3-dibromo-4,5-dihydroxy-phenyl)-methane, in the proliferation, migration and invasion of hepatocellular carcinoma cells (BEL-7402). Exhibit a IC_50_ value of 15.9 μM and, at concentration of 9.1 µM, the cell adhesion to fibronectin and collagen IV as well as cell migration and invasion decrease significantly, while at 18.2 µM a complete inhibition of the migration and MMP-2 and MMP-9 expression occurs. Moreover, compound **47** inhibits the focal adhesion kinase (FAK), a protein required for cell transformation and invasion [145]. The compound **47** also inhibits multiple angiogenesis processes, including endothelial cell sprouting, migration, proliferation, and tube formation. It seems to be a potent and selective inhibitor of the tyrosine kinase receptor and exhibiting multi effects of inhibition. Moreover, at 10 μM inhibit the activities of FGFR2 and FGFR3, VEGFR2 and PDGFRα factors and decreases the phosphorylation of PKB/Akt and eNOS, as well as the NO production [146].

The inhibitory effect of compounds **48** and **49** against the protein tyrosine kinase (PTK) with over–expression of the proto-oncogene *c-kit* was evaluated and the results showed significant inhibition ratio of this protein (80.1% and 71.4% respectively) [147].

In 2012, Liu et al. [148] selected the compound **50** (bis(2,3-dibromo-4,5-dihydroxybenzyl) ether), a cytotoxicity compound against a wide range of cancer cell lines (IC_50_ ≤ 9 µM, Table 3), for detailed analysis of its effect on K562 cell line. The authors demonstrated that the mitochondrial pathway was involved in the bromophenol **50** induced apoptosis. In fact, bromophenol **50** induces S phase arrest and inhibits topoisomerase I activity followed by apoptosis. It is interesting to mention that compound **50**, contrary to the mechanism of the topoisomerase I inhibitor campthotecin, was not able to stimulate the formation of topoisomerase I–DNA complex or able to intercalate DNA. These facts suggest a different inhibition mechanism [148]. Considering that the current topoisomerase I inhibitors exhibit significant side effects [149] and the uniqueness of compound **50** structure, its significant cytotoxic activity against a broad range of cancer cell lines coupled with its distinct mechanism of action, indicate that compound **50** may, undoubtedly, be a leading compound for the development of a new generation of anticancer topoisomerase I inhibitors.

Qi et al. also studied the bromophenol **50** effects on angiogenesis and detected that it can significantly repress the human umbilical vein endothelial cells (HUVEC) cells proliferation without induced apoptosis. Can also decrease migration and tube formation, without effect on the performed vascular tube, via inhibiting VEGF signal systems [150].

### 2.4. Alkaloids

Alkaloids isolated from seaweeds include indoles and indoles analogues, quinone, 2-phenylethylamine and 2,7-naphthyridine derivatives, however the most common ones belong to the indole and 2-phenylethylamine groups [151]. The indole type is concentrated in Rhodophyta (red seaweeds) while halogenated alkaloids, mainly bromine- and chloride-containing, are dominant in Chlorophyta (green seaweeds) [152]. Indole type alkaloids are also important because they represent a quarter of all alkaloids that are regarded as promising compounds in the development of new drugs.

**Figure 6:**
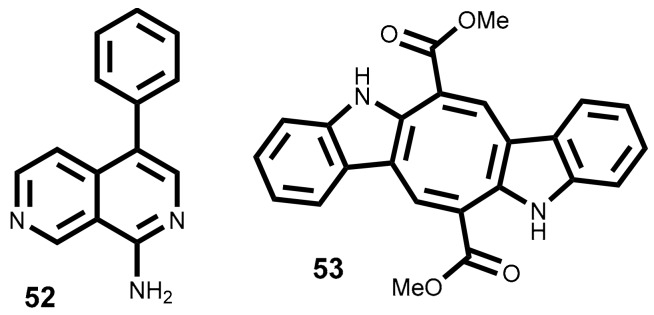
Chemical structure of cytotoxic alkaloids isolated from seaweeds.

The lophocladine B **52** (Figure 6) is a 2,7-naphthyridine alkaloid isolated from red alga *Lophocladia* sp. collected in the Fijan Islands [153]. The cytotoxicity of this molecule was evaluated against NCI-H460 (lung cancer cell line) and MDA-MB-435 (used by the authors as a human breast cancer model but currently considered as a human melanoma model [154]). Lophocladine B **52** exhibits significant cytotoxic effect against and MDA-MB-435 cell line (IC_50_ = 3.1 µM), causing a major reduction of cells in the G1 and S phases with G2/M cell cycle arrest [153]. Moreover, the analyses of lophocladine B **52** effects on cellular microtubes and actin filaments in A-10 cells (used as vascular smooth muscle cells model) showed that this metabolite acts as microtubules inhibitor with no loss of actin filament [153]. In the same year Lotter et al. reported that lophocladine B **52** is also able to inhibit human leukemia cells (HL-60) (IC_50_ = 1 µM) [155].

The well-known potential of the antitubulin compounds as effective anticancer agents, for example taxol and vincristine, spread the interest of the scientific community in the synthesis of lophocladine B **52** or derivatives, seeking new and more potent antitumor agents and/or structure activity relationship studies [155,156,157,158]. To date, only two of the synthesized compounds (simplified isoquinolines isoquinoline-triazole derivatives) exhibit better cytotoxicity (IC_50_ = 10.14 and 12.76 µM) than lophocladine B **52** against hepatic cancer (HepG2) and cervical cancer (HeLa) cell lines (IC_50_ = 89.5 and >100 µM) [158]. However, the synthesis of lophocladine B **52** allowed the conclusion that the cytotoxic level depends on the C1 substituent. For example, *tert*-amine is less potent than primary- and secondary-amines [157].

Indole alkaloids have exhibited a wide array of biological activities and the frameworks produced by marine species are often very different from those produced by terrestrial species [159,160,161]. One interesting example is the bisindole caulerpin or caulerpine **53** (Figure 6), isolated mainly from green algae genus *Caulerpa* [161,162] but also from *Codium decorticatum* (Woodward) M. Howe [163] and *Halimeda incrassata* (J. Ellis) J.V. Lamouroux [164]. And also found in some red algae such as *Chondria armata* (Kützing) Okamura [165], *Laurencia dendroidea* J. Agardh [syn. *Laurencia majuscula* (Harvey) A.H.S. Lucas] and *Caloglossa leprieurii* (Montagne) G. Martens [166].

Some of the abovementioned seaweeds are invasive species and the study of their chemical entities can contribute to suggesting a possible use for that biomasses, being the isolation of valuable chemicals one of the most explored uses. In this context, caulerpin **53** is among the most studied metabolites. Unfortunately, caulerpin **53** effects on cell growth/viability are not very interesting, IC_50_ values ≥ 20 µM against some cancer cell lines (T47-D, MCF-7, MDA-MB-231, PC3 DU145, HMEC, HCT116, HT29, LOVO and SW480) [167,168].

Even though, caulerpin **53** action mechanism was studied and it was proved that caulerpin **53** inhibits hypoxia-inducible factor 1 (HIF-1), an important target in cancer therapy [167]*.* The results suggested that caulerpin **53** at concentration of 10 µM and under hypoxic conditions blocked the induction of HIF-1α protein, the HIF-1 oxygen-regulated subunit, while at concentration of 30 µM, it disrupts mitochondrial ROS-regulated HIF-1 activation and downstream HIF-1 pathway [167]. Caulerpin **53** also exhibits effect on tumor cell migration once it suppressed the migration of metastatic MDA-MB-231 cells in a concentration-dependent manner, being the better results observed at 30 µM [167].

Caulerpin **53** overpowers the mitochondrial respiratory at complex II and increases ROS production in cisplatin-resistant C13 ovarian cancer cells, while it has no effect on the complexes I, III and IV [169], suggesting that some derivatives could be good candidates for the treatment of cisplatin-resistant cancer, once ovarian cancer cisplatin resistant cells are characterized by reduced oxygen consumption and increased dependence on glucose [170].

More recently, the anticancer effect of caulerpin **53** against LOVO cancer cell line was evaluated, and the results showed a cytotoxic effect with IC_50_ = 20 µM [168]. The mechanism of action study indicates that caulerpin **53** inhibits oxidative phosphorylation (OXPHOS), that is inhibits the metabolic pathway in which cells use enzymes to oxidize nutrients to release energy. LOVO cells are one example of those cells that are less glycolytic and more depended on OXPHOS to produce adenosine triphosphate (ATP). It was also demonstrated that in combination therapy, caulerpin **53** can be used with the glycolytic inhibitor 3-bromopyruvate to prevent LOVO cellular proliferation [168].

## 3. Secondary Metabolites from Seaweeds with In Vivo Antitumor Activity

Some of the secondary metabolites mentioned in the previous section were subjected to in vivo evaluations, not as much as it is recommended. Those studies, due to their significance, will be herein presented and discussed.

Preliminary in vivo evaluation of halomon **33** (Figure 1) showed that daily treatments (5× doses of 50 mg/kg) in an ip/ip xenograft model with highly aggressive U251 brain cancer showed 40% of success. However, research was stopped due to insufficient amount of halomon **33** to perform more essential tests. Both attempts to extract more material from the natural sources and development of efficient synthetic procedures demonstrate that the amount obtained could not be use by pharmaceutical industry [57].

The in vivo inhibitory effect of fucoxanthin **38** (Figure 3) in duodenal carcinogenesis was demonstrated using mice with duodenal carcinogenesis, which was induced by *N*-ethyl-*N*’-nitro-*N*-nitrosoguanidine. The mice were treated with fucoxanthin **38** [oral administration of 0.005% solution of fucoxanthin in drinking water], during 4 and 16 weeks. This in vivo study demonstrates that fucoxanthin **38** inhibits ornithine decarboxylase (ODC) activity [171], an enzyme whose activity is induced in response to cell growth stimuli and is highly expressed in diseases such as inflammation and cancer being not only a biomarker for cancer but also a potential target for its therapy [172].

Fucoxanthin **38** was also evaluated on melanin synthesis in vivo and was found that topical application (once a day to the back skin of the mice shortly after UVB irradiation) of an ointment (mixture of fucoxanthin at 0.01, 0.1 and 1% in white petrolatum) and oral treatment [fucoxanthin at 0.1, 1 and 10 mg/kg b.w. in water with 5% acacia, given once a day, 2 h before UVB irradiation (160 mJ/cm^2^) for seven days followed by increased UVB (320 mJ/cm^2^) irradiation for seven more days], suppressed skin mRNA expression related to melanogenesis [173]. Identical effect was observed on UV-induced skin pigmentation in guinea-pigs [173]. In conclusion these results suggest that fucoxanthin **38** exhibits in vivo anti-pigmentary activity, in UVB-induced melanogenesis, by topical or oral application. This effect of fucoxanthin **38** may be due to suppression of prostaglandin (PG) E2 synthesis and melanogenic stimulant receptors (neurotrophic, PGE2 and melanocyte stimulating hormone expression) [173].

Finally, fucoxanthin **38** at 25, 50 and 100 mg/kg b.w. oral administration dose, once per day for 1 week, exhibits the ability to induce apoptosis of sarcoma 180 (S180) [174]. The in vivo study was performed using male Kunming mice and the results indicated that treatment with fucoxanthin **38** remarkably reduce the expression of EGFR in the tumor tissue when compared with the model control group, suggesting that down-regulation of STAT3/EGFR signaling appeared to be involved in the in vivo anti-tumor effect and apoptosis induction [174].

Bald/c athymic female nude mouse with ovarian carcinoma induced by subcutaneously inoculating 5 × 10^6^ SKOV3 cells, were used for in vivo studies of anticancer effects of dieckol **41** (Figure 4). For 4 consecutive weeks, the experimental group of mice is treated with dieckol **41** (50 and 100 mg/kg b.w.) or cisplatin (3 mg/kg b.w.) three times per week [131]. The results demonstrated that dieckol **41** suppressed tumor growths compared with the control group without any significant adverse effect in the SKOV3-bearing mouse model [131].

In 2017, the efficacy of dieckol **41** (oral admistration of 40 mg/kg b.w. dose for 15 weeks) as anticancer against *N*-nitrosodiethylamine (NDEA)-induced hepatocarcinogenesis in male albino Wistar rats was evaluated [175]. The treatment with dieckol **41** caused the suppression of the NDEA-initiated hepatocarcinogenesis by modulation of xenobiotic-metabolizing enzymes with modulation of cell proliferation and induction of apoptosis via the mitochondrial pathway. The inhibition of invasion and angiogenesis also observed was evidenced by the decrease of both MMP-2 and MMP-9 activities as well as the decrease of the vascular endothelial growth factor (VEGF) expression. Dieckol **41** also exerts anticancer effects via inhibition of the pro-inflammatory transcription factors NF-ĸb and COX2 [175].

Phloroglucinol **39** (Figure 4) (at 25 mg/kg b.w. dose administrated four times at alternate days) suppresses colony formation and in vivo tumorigenicity in BT549 breast cancer cells [132]. Tumor were formed by subcutaneous inoculation of 1 × 10^6^ sphere-cultured BT549 breast cancer cells into athymic Bald/c female nude mice and phloroglucinol **39** decreased dramatically the tumor volume, suggesting that this secondary metabolite inhibits tumorigenic capacity by targeting cancer stem-like cells and/or non-cancer stem-like cells (CSCs) [132].

To study the phloroglucinol **39** effect on metastatic breast cancer (MDA-MB-231) in vivo, the model metastatic MDA-MB-231 cells injected into fourth mammary fat pad of NOD scid gamma (NSG) mice was used [133]. The treatment with phloroglucinol **39** (25 mg/kg b.w.) administrated four times on alternate days, attenuated the primary tumor formation in mammary fat pads, and attenuated the lung metastasis and the expression levels of vimentin (VIM) and SLUG proteins [133]. Similarly, to the in vitro data, treatment with phloroglucinol **39** decreased phosphorylation of AKT and ERK, consequently treated mice survive longer than the control mice [133].

The in vivo study to evaluate the activity of bromophenol **50** (Figure 5) was carry on zebrafish model, an ideal model used in screening of anti-angiogenic agents. At a concentration of 25 µM, bromophenol **50** inhibited the sub-intestinal vessel (SIV) formation in 49.5%, confirming it inhibitory activity against angiogenesis. The results also suggest that bromophenol **50** can, in concentration dependent manner, produce toxic effects towards the zebrafish development [150].

Our final example is the in vivo effect of caulerpin **53** (Figure 6), in combination with 3-bromopyruvate, on athymic nude mouse model bearing SW480 implanted xenografts. The combination therapy of 3-bromopyruvate and caulerpin **53** (30 mg/kg b.w.) displayed remarkable tumor regression. Complementary analysis demonstrated that proliferating cell nuclear antigen (PCNA) and phosphorylated mammalian target of rapamycin (p-mTOR) expression was significantly inhibited in this combined therapy, showing the key role of adenosine monophosphate-activated protein kinase (AMPK)/mTOR pathway in the anticancer activity of caulerpin **53** [168].

## 4. Conclusions

In this review the in vitro activities of 53 secondary metabolites isolated from brown, red and green seaweeds are presented and discussed. The mechanisms of action, structure/activity relationship and, in the cases that was possible, the in vivo studies were also discussed. From literature published in the last 30 years, were selected the secondary metabolites that exhibit cytotoxic activity against tumor cell lines with IC_50_ values lower than 15 µM. The compounds reported belong mainly to the terpenoids class, followed in much smaller number by compounds of bromophenols class, phlorotannins and alkaloids.

The diterpene **11** (dictyolactone), the sterol **22** (cholest-5-en-3β,7α-diol) and the halogenated monoterpene **33** (halomon) are the only reported compounds with sub-micromolar activity (IC_50_ ≤ 0.9 µM) against at least one cancer cell line, while laurebiphenyl **20**, a dimeric sesquiterpene of the cyclolaurane-type, and the halogenated monoterpene **31**, [(1*E*,5*E*,7*E*)-3,4-*erythro*-1-bromo-7- -dichromethyl-3-methyl-3,4,8-trichlorooctatriene] are relevant because they exhibit IC_50_ values between 1–5 µM against 6 cell lines (lung, cervix, gastric, hepatic, ileum, colon cancer cell lines). Of the remaining compounds, it is noteworthy the bromophenol **51**, which exhibits high cytotoxic activity (IC_50_ ≤ 3.8 µM) against the lung, gastric, breast, colon, melanoma and ovarian cancer cell lines, the bis (2,3-dibromo-4,5-dihydroxy-phenyl)-methane **47** with identical cytotoxic activity against the first 4 tumoral cell lines and *n*-propyl ether lanosol **46** the most active bromophenol (IC_50_ = 1.32 µM against colon cancer cell line), in fact more active than the 5-fluorouracil. These are, as far as we could find, the most promising secondary metabolites isolated from seaweeds and reported in the literature.

It should be emphasized that most of the promising compounds outlined herein belong to classes of secondary metabolites that are not biosynthesized in terrestrial species, being the most evident ones the halogenated terpenes and bromophenols. This fact reinforces the importance of the marine environment in general and the seaweeds in particular as a source of potentials new anticancer drugs.

Nevertheless, several studies remain to be performed. Only a few compounds (only 9 out of 53 presented) were subjected to selectivity studies, and none of them correspond to the most promising ones. Also, the in vivo studies are scarce (5 out of 53 cytotoxic compounds reviewed), except for fucoxanthin **38**, dieckol **41** and phloroglucinol **39**. But this compound advanced studies can be explained because they are already recognized as having high pharmacological potential for the prevention/treatment for example cardiovascular diseases [20,25].

An added value of these compounds that can be detected by this literature survey is clearly shown by the in vitro and in vivo works demonstrating different mechanisms of action and targets. These facts suggest that the seaweeds’ secondary metabolites may be used to develop new anticancer agents with distinct ways of action.

Surprisingly, the most advanced studies on mechanisms of action and/or in vivo studies were not performed with the more promising compounds. Most likely because they are scarce in nature and/or their synthesis is not yet accomplished. Even so, we believe that some of the above-discussed compounds exhibit activities that justify greater attention from the scientific community.

## Figures and Tables

**Table 1:** Cytotoxic terpenoids isolated from seaweeds with half maximal inhibitory concentration (IC_50_) lower than 15 µM against tumoral cell lines.

Metabolites	Sources ^a^ [ref.]	Cell Lines Tested (IC_50_ Value µM) [ref.]
**1**	*Cystoseira myrica* (S.G.Gmelin) C.Agardh) [32]	KA3IT (13.1); NIH3T3 ^b^ (19.7) [32]
**2**	*Cystoseira myrica* (S.G.Gmelin) C.Agardh [32]	KA3IT (13.7); NIH3T3 ^b^ (20.6) [32]
**3**	*Cystoseira myrica* (S.G.Gmelin) C.Agardh [32]*Dictyota dichotoma* (Hudson) J.V.Lamouroux [33]	KA3IT (14.4); NIH3T3 ^b^ (43.3) [32]
**4**	*Cystoseira abies-marina* (S.G. Gmelin) C. Agardh [34]	HeLa–Log phase (5.6); Vero ^b^–Log phase (14.6) [34]
**5**	*Dilophus ligulatus* (Kützing) Feldmann [35]	P388 (4.33–4.79) [35]
**6**	*Dilophus ligulatus* (Kützing) Feldmann [35]	P388 (11.2); P388/DOX (12.9); NSCLCN6-L16 (6.61) [35]
**7**	*Dilophus ligulatus* (Kützing) Feldmann [35]	P388 (3.64); P388/DOX (5.95); KB (12.2); NSCLCN6-L16 (3.31) [35]
**8**	*Dilophus ligulatus* (Kützing) Feldmann [35]	P388 (7.42); P388/DOX (9.41); KB (14.3), NSCLCN6-L16 (6.85) [35]
**9**	*Dilophus ligulatus* (Kützing) Feldmann [35]	P388 (10.3); P388/DOX (10.8) KB (6.85); NSCLCN6-L16 (1.71) [35]
**10**	*Dilophus ligulatus* (Kützing) Feldmann [35]	P388 (13.3); KB (14.4); NSCLCN6-L16 (4.44) [35]
**11**	*Dilophus ligulatus* (Kützing) Feldmann [35]	P388 (9.26); P388/DOX (7.93) NSCLCN6-L16 (0.99) [35]
**12**	*Stypopodium zonale* (J.V.Lamouroux) Papenfuss [36]	HT-29 (5.83); H-116 (5.83); A549 (5.83) [36]
**13**	*Stypopodium flabelliforme* Weber-van Bosse [37]	NCI-H460 (9) [37]
**14**	*Stypopodium flabelliforme* Weber-van Bosse [37]	NCI-H460 (14) [37]
**15** and **16**	*Taonia atomaria* (Woodward) J. Agardh [38]	NSCLC-N6 (7.35); A549 (7.35) [38]
**17**	*Stypopodium flabelliforme* Weber-van Bosse [39]	SH-SY5Y (12.2) [39]
**18**	*Stypopodium flabelliforme* Weber-van Bosse [39]	SH-SY5Y (14) [39]
**19**	*Sargassum fallax* Sonder [40]*Sargassum yezoense* (Yamada) Yoshida & T. Konno [41]	P388 (14) [40]
**20**	*Laurencia tristicha* Tseng, Chang, E.Z. et B.M. Xia [42]*Laurencia nidifica* J. Agardh [43]	A549 (3.94); HeLa (3.77); BGC-823 (2.86); Bel7402 (4.48); HCT-8 (4.15) [42]
**21**	*Laurencia tristicha* Tseng, Chang, E.Z. et B.M. Xia [44]	HeLa (15.5) [44]
**22**	*Laurencia tristicha* Tseng, Chang, E.Z. et B.M. Xia [44]	HeLa (0.3); BGC-823 (5.1); Bel7402 (0.5); HCT-8 (0.5); HT29 (9.1) [44]
**23**	*Laurencia pacifica* Kylin [45]*Laurencia okamurae* Yamada [46]	DU145 (6.8); HT-29 (9.1); A431 (9.6); A2780 (10); BE2-C (13); MCF-7 (14); SMA (14); SJ-G2 (15); MCF10A ^b^ (28) [45]
**24**	*Laurencia pacifica* Kylin [45]*Laurencia okamurae* Yamada [46]	HT-29 (15); DU145 (12); MCF10A ^b^ (46) [45]
**25**	*Plocamium suhrii* Kützing [47]*Plocamium cartilagineum* (Linnaeus) P.S.Dixon [48,49]	WHCO1 (6.6) [47]; CFU (3.36) [48]
**26**	*Plocamium suhrii* Kützing [47]*Plocamium cartilagineum* (Linnaeus) P.S.Dixon [48,49]	WHCO1 (9.9) [47]
**27**	*Plocamium suhrii* Kützing [47]	WHCO1 (9.3) [47]
**28**	*Plocamium suhrii* Kützing [47]*Plocamium cartilagineum* (Linnaeus) P.S.Dixon [49] *Plocamium oregonum* Doty [50]	WHCO1 (8.5) [47]
**29**	*Plocamium suhrii* Kützing [47]*Plocamium corallorhiza* (Turner) Hooker & Harvey [51]	WHCO1 (7.9) [47]
**30**	*Plocamium suhrii* Kützing [47]	WHCO1 (8.4) [47]
**31**	*Plocamium cartilagineum* (Linnaeus) P.S.Dixon [48,49]	CFU (3.36) [48] HCT-116 (3.36) [52]
**32**	*Plocamium cartilagineum* (Linnaeus) P.S.Dixon [48]	NCI-H460 (13) [48]
**33**	*Portieria hornemannii* (Lyngbye) P.C.Silva [53]	HCT-116 (0.92) [52,53]

^a^ In this table are indicated the seaweed Latin botanical name used by the authors, which, according to the database AlagaeBase (http://www.algaebase.org), does not always correspond to a currently accepted name; ^b^ No-tumoral cell line; A2780 = Ovarian cancer cell line; A431 = Human skin carcinoma cell line; A549 = human alveolar basal epithelial adenocarcinoma cell line; BE2-C = Neuroblastoma cancer cell line; Bel7402 = Hepatocellular carcinoma; BGC-823 = Human gastric cancer cell line; CFU = human colon cancer cell line; DU 145 = Human prostate carcinoma cell line; HCT-8 = Epithelial human ileum cell line; HCT 116 = Human colon carcinoma; HeLa = Cervix adenocarcinoma cell line; HT-29= Human colon cancer cell line; KA3IT = Virally transformed form mouse cancer cell line; KB = Human nasopharynx carcinoma; MCF 10A = Human mammary epithelial normal cell line; NCI-H460 = Human lung carcinoma cell line; NIH3T3 = Cell line murine, fibroblast; NSCLC-N6 = Squamous cell lung carcinoma; NSCLCN6-L16 = Human non-small cell lung carcinoma cells; P388 = Mouse leukemia cell line; P388/DOX = Doxorubicin resistant mouse leukaemia cell line; SH-SY5Y = Human bone marrow neuroblastoma cancer cell line; SJ-G2 = Human glioblastoma cancer cell line; SMA = Murine glioblastoma cancer cell line; Vero = Kidney no-tumoral cell line; WHCO1 = Oesophageal cancer cell line.

**Table 2:** In vitro cytotoxic activity and action mechanism of fucoxanthin isolated from seaweeds.

Fucoxanthin Source	Cell Line Tested	Level of Activity	Activity/Mechanism	Target Molecules	Ref.
*Hizikia fusiformis* (Harvey) Okamura	GOTO	38% growth inhibition at 15.2 µM	G1 cell cycle arrest	N-myc	[84]
*Undaria pinnatifida* (Harvey) Suringar	HL-60	46% cellular viability at 11.3 µM	Apoptosis induction	Caspase-3; caspase-7; caspase-9	[85]
Unspecified source	PC3, DU145, LNCap	At 20 µM cellular viability was 14.9% of PC3, 5% of DU145 and 9.8% of LNCap cell lines	Apoptosis induction	Bcl-2; Bax; caspase-3	[86,87]
*Undaria pinnatifida* (Harvey) Suringar	CaCo-2, DLD-1, HT29	36.8% cellular viability of CaCo-2 at 7.6 µM	Apoptosis and antiproliferative effect	Bcl-2	[88]
*Laminaria japonica* Areschoug	WiDr, HCT116	At 25 µM cell cycle arrest and phosphorylation of pRb	Cell cycle arrest G_0_/G_1_	P21^WAF1/Cip1^	[71]
Unspecified source	HepG2, DU145	IC_50_ = 3 µM	Cell cycle arrest G_1_	GADD45A	[89]
*Laminaria japonica* Areschoug	HepG2	At 25 µM, inhibition in 29.5% of cell proliferation	Cell cycle arrest G_1_	Cyclin D	[90]
*Laminaria japonica* Areschoug	EJ-1	At 6.25 µM the cellular viability decreased to 52.62%	Apoptosis induction	Caspase-3	[91]
*Undaria pinnatifida* (Harvey) Suringar	SK-Hep-1	IC_50_ 9.4 µM	Cell cycle arrest G1, apoptosis induction	Connexin 43; connexin-32	[92]
*Ishige okamurae* Yendo	HL-60	Inhibition of proliferation in 65% at 15 µM	Apoptosis induction	Caspase-3; caspase-7; PARP; Bcl-xL	[93]
*Undaria pinnatifida* (Harvey) Suringar	MGC-803	At 75 µM apoptosis inducing effects like Paclitaxel (1 µM)	Cell cycle arrest G2/M, apoptosis induction	CyclinB1; survivin	[94]
Unspecified source	LNCap	IC_50_ = 2.5 µM	Cell cycle arrest G_1_	GADD45A; SAPK/JNK	[95]
*Undaria pinnatifida* (Harvey) Suringar	HUVEC	At 5 µM inhibition of FGF-2 expression in 22%	Inhibition of angiogenesis	FGF-2; FGFR-1	[96]
Unspecified source	T24	Induced apoptosis at 40 µM and proliferation inhibition at 5–10 µM	Apoptosis induction; Cell cycle arrest G1	Caspase-3; cyclin D1; cyclin E	[97]
Unspecified source	U87, U251	At 25 µM the cellular viability decreased 52% and 43% in U87 and U251, respectively	Apoptosis induction. Inhibition of invasion and migration	Caspase-3; caspase-9; cleaved-PARP; P38-MMP-2/9	[98]
Unspecified source	SiHa	Strong synergistic effect from combination with TRAIL	Apoptosis induction	Caspase-3; Bcl-2; Bax	[99]
*Undaria pinnatifida* (Harvey) Suringar	SGC7901	IC_50_ = 9.80 ± 0.94 (48 h)	Apoptosis and autophagy induction	Beclin-1; caspase-3; Bcl-2	[100]

Bcl-xL = B-cell lymphoma extra-large; CaCo-2 = Human colon adenocarcinoma cell line; DLD-1 = Human colon adenocarcinoma cell line; DU-145 = Human prostate carcinoma cell line; EJ-1= Human lymphoma; FGF = Fibroblast growth factors; GOTO = Human neuroblastoma cell line; HepG2 = Human liver hepatocarcinoma cell line; HCT-116 = Human colon carcinoma cell line; HL-60 = Human leukaemia cell line; HT-29 = Human colorectal adenocarcinoma cell lines; HUVEC = Human umbilical vein endothelial cells; LNCap = Human prostate carcinoma cell line; MGC-803 = Human gastric carcinoma cell line; N-myc = Proto-oncogene protein; P21^WAF1/Cip1^ = Cyclin-dependent kinase inhibitor 1; PARP = poly-ADP-ribose polymerase; PC-3 = Human prostate adenocarcinoma cell line; SAPK/JNK = c-Jun N-terminal kinases; SiHa = Human cervix uteri cell line; SGC7901 = Human gastric cancer cell line; SK-Hep-1 = Human endothelial adenocarcinoma cell line; T24 = Human bladder carcinoma cell line; U87 and U251 = Human primary glioblastoma cell line; WiDr = Human colorectal adenocarcinoma cell lines.

**Table 3:** Bromophenols with potential cytotoxic activity isolated from seaweeds.

Metabolites	Sources	Cell Lines Tested (IC_50_ Value µM)
**42**	*Vertebrata lanosa* (Linnaeus) T.A. Christensen [137], *Neorhodomela larix* (Turner) Masuda [134,140], *Odonthalia corymbifera* (S.G. Gmelin) Greville [135]	DLD-1 (14.6); HCT-116 (14.1) [137]
**43**	*Vertebrata lanosa* (Linnaeus) T.A.Christensen [137], *Osmundaria serrata* (Suhr) R.E. Norris [138]	DLD-1 (13.5); HCT-116 (2.51) [137]
**44**	*Vertebrata lanosa* (Linnaeus) T.A. Christensen [137]	DLD-1 (12.4); HCT-116 (1.32) [137]
**45**	*Rhodomela confervoides* (Hudson) P.C. Silva [141]	KB (12.5); Bel7402 (12.9); A549 (14.4); HELF ^a^ (25.9) [141]
**46**	*Leathesia marina* (Lyngbye) Decaisne [142,143]	A549 (2.5); BGC823 (8.8); MCF-7 (2.7); Bel7402 (4.8); B16-BL6 (7.3); HT-1080 (6.6); A2780 (2.7) [142,143]
**47**	*Leathesia marina* (Lyngbye) Decaisne [142], *Rhodomela confervoides* (Hudson) P.C. Silva [144]	A549 (1.8); BGC823 (3.8); MCF-7 (2.7); HCT-8 (2.2) [142]
**48**	*Leathesia marina* (Lyngbye) Decaisne [142,143]	BGC823 (4.6); MCF-7(3.4); Bel7402 (5.5); HCT-8 (2.8); B16-BL6 (3.3), HT-1080 (7.2); A2780 (7.1) [142,143]
**49**	*Leathesia marina* (Lyngbye) Decaisne [142,143]	BGC823 (8.6); B16-BL6 (15.4); HT-1080 (10.3) [142,143]
**50**	*Leathesia marina* (Lyngbye) Decaisne [142,143]	A549 (5.4); MCF-7 (4.6); Bel7402 (7.4); HCT-8 (5.9); HT-1080 (8.2); A2780 (8.6) [142,143]
**51**	*Leathesia marina* (Lyngbye) Decaisne [143]	A549 (1.6); BGC823 (3.3); MCF-7 (2.5); HCT-8 (1.9); B16-BL6 (3.2); A2780 (3.8) [143]

^a^ No-tumoral cell line A2780 = Ovarian carcinoma cell line; A549 = Human lung carcinoma cell line; B16-BL6 = Murine melanoma cell line; Bel7402 = Hepatocellular carcinoma cell line; BGC-823 = Human gastric cancer cell line; DLD-1 = Human colon adenocarcinoma cell line; HCT-8= Human colon adenocarcinoma cell line; HCT-116 = Human colon carcinoma cell line; HELF = Non-tumoral human embryo lung fibroblasts cell line; HT-1080 = Human fibrosarcoma cell line; KB = Human nasopharynx carcinoma; MCF-7= Human breast adenocarcinoma cell line.

## Abbreviations

Akt	Serine/Threonine-Specific Protein Kinase
AMPK	Adenosine Monophosphate-Activated Protein Kinase
ATP	Adenosine Triphosphate
BALB	“Bagg Albino”
Bax	B-Cell Lymphoma 2-Associated X Protein
BCBL-1	Body-Cavity-Based Lymphoma Cell Line
BCL-xL	B-Cell Lymphoma-Extra Large
BT549	Human Carcinoma Breast Epithelial Cell Line
b.w.	Body Weight
CD44	Cluster of Differentiation 44
cdc2	Cell Division Cycle Protein 2
CDK4	Cyclin-Dependent Kinase 4
CpG	Cytosine Nucleotide Followed by A Guanine Nucleotide
CIAP	Cellular Inhibitors of Apoptosis Protein
COX	Cyclooxygenase
CSCs	Cancer Stem-Like Cells
CTX	Cyclophosphamide
CXCR4	CXC Motif Chemokine Receptor 4
DMSO	Dimethyl Sulphoxide
DNA	Deoxyribonucleic Acid
DNMT-1	DNA Methyltransferase-1
EGFR	Epidermal Growth Factor Receptor
eNOS	Endothelial Nitric Oxide Synthase
ERK	Extracellular Signal–Regulated Kinase
FAK	Focal Adhesion Kinase
FGFR	Fibroblast Growth Factor Receptor
G1 phase	Gap 1 Phase
GTP	Guanosine-5′-triphosphate
HaCaT	Aneuploid Immortal Keratinocyte Cell Line
HIF-1α	Hypoxia-Inducible Factor 1-Alpha
IC_50_	The Half Maximal Inhibitory Concentration
K562	Human Chronic Myelogenous Leucemia Cell Line
LOVO	Human Colon (Supraclavicular Lymph Node Metastasis)
M	Mitosis
MAPK	Mitogen-Activated Protein Kinase
MDA-MB-231	Human Adenocarcinoma Breast Cell Line
MMP	Matrix Metalloproteinase
mRNA	Messenger Ribonucleic Acid
MYCN	Myelocytomatosis
NDEA	*N*-nitrosodiethylamine
NF-ĸB	Nuclear Factor-kappa B
NOD	Nonobese Diabetic
Notch-2	Neurogenic Locus Notch Homolog Protein-2
NSG	NOD Scid Gamma
Oct-4	Octamer-Binding Transcription Factor 4
ODC	Ornithine Decarboxylase
OXPHOS	Oxidative Phosphorylation
p15^INK4B^	Cyclin-Dependent Kinase Inhibitor 4B
p21^WAF1/CIP1^	Cyclin-Dependent Kinase Inhibitor 1
p27^KIP1^	Cyclin-Dependent Kinase Inhibitor 1B
p75NTR	Neurotrophin Receptor p75
PARP	Poly (ADP-Ribose) Polymerase
PCNA	Proliferating Cell Nuclear Antigen
PDGFRα	Platelet-Derived Growth Factor Receptor α
p-mTOR	Phosphorylated Mammalian Target of Rapamycin
PI3K	Phosphoinositide 3-Kinase
PTK	Protein Tyrosine Kinase
RAF-1	Murine Leukemia Viral Oncogene Homolog 1
RAS	Rat Sarcoma
Rb	Retinoblastoma
ROS	Reactive Oxygen Species
S phase	Synthesis Phase
SCID	Severe Combined Immunodeficiency
Ser	Serine
SIV	Sub-Intestinal Vessel
SLUG	Human Embryonic Protein Snail Family Transcriptional Repressor 2
Sox2	(Sex Determining Region Y)-Box 2
STAT3	Signal Transducer and Activator of Transcription 3
SW480	Human Colorectal Adenocarcinoma Cell Line
TIMP	Tissue Inhibitor of Metalloproteinase
TRADD	Tumour Necrosis Factor Receptor Superfamily Member 1A Associated via Death Domain
TY-1	Primary Effusion Lymphoma Cell Line
U251	Human Brain Glioblastoma Cell Line
UVB	Ultraviolet Radiation B
VEGFR2	Vascular Endothelial Growth Factor Receptor 2
VIM	Vimentin
XIAP	X-Linked Inhibitor of Apoptosis Protein
